# Nosocomial Outbreak of Novel Arenavirus Infection, Southern Africa

**DOI:** 10.3201/eid1510.090211

**Published:** 2009-10

**Authors:** Janusz T. Paweska, Nivesh H. Sewlall, Thomas G. Ksiazek, Lucille H. Blumberg, Martin J. Hale, W. Ian Lipkin, Jacqueline Weyer, Stuart T. Nichol, Pierre E. Rollin, Laura K. McMullan, Christopher D. Paddock, Thomas Briese, Joy Mnyaluza, Thu-Ha Dinh, Victor Mukonka, Pamela Ching, Adriano Duse, Guy Richards, Gillian de Jong, Cheryl Cohen, Bridget Ikalafeng, Charles Mugero, Chika Asomugha, Mirriam M. Malotle, Dorothy M. Nteo, Eunice Misiani, Robert Swanepoel, Sherif R. Zaki, members of the Outbreak Control, Investigation Teams

**Affiliations:** National Institute for Communicable Diseases, Sandringham, South Africa (J.T. Paweska, L.H. Blumberg, J. Weyer, G de Jong, C. Cohen, R. Swanepoel); University of the Witwatersrand, Johannesburg, South Africa (N.H. Sewlall, M.J. Hale, A. Duse); Centers for Disease Control and Prevention (CDC), Atlanta, Georgia, USA (T.G. Ksiazek, S.T. Nichol, P.E. Rollin, L.K. McMullan, C.D. Paddock, T.-H. Dinh, S.R. Zaki); Columbia University, New York, New York, USA (W.I. Lipkin, T. Briese); Gauteng Department of Health, Johannesburg (J. Mnyaluza, B. Ikalafeng, C. Asomugha); CDC Global AIDS Program, Pretoria, South Africa (T.-H. Dinh); Ministry of Health, Lusaka, Zambia (V. Mukonka); CDC Global AIDS Program, Lusaka (P. Ching); Charlotte Maxeke Hospital, Johannesburg (G. Richards); National Department of Health, Pretoria (C. Mugero, E. Misiani); Field Epidemiology and Laboratory Training Programme, Johannesburg (M.M. Malotle, D.M. Nteo); University of Texas Medical Branch, Galveston, Texas, USA (T.G. Ksiasek)

**Keywords:** Arenavirus, Lujo virus, nosocomial infections, South Africa, Zambia, viruses, research

## Abstract

A nosocomial outbreak of disease involving 5 patients, 4 of whom died, occurred in South Africa during September–October 2008. The first patient had been transferred from Zambia to South Africa for medical management. Three cases involved secondary spread of infection from the first patient, and 1 was a tertiary infection. A novel arenavirus was identified. The source of the first patient’s infection remains undetermined.

## Abstract 2

This case reinforces the need for strict screening of internationally transferred patients.

Arenaviruses associated with rodents are known to cause fatal hemorrhagic fevers in humans in South America and West Africa. We describe a nosocomial outbreak of infection with a novel arenavirus involving 5 patients, 4 of whom died, which occurred in South Africa in September–October 2008. The first patient was transferred from Zambia to South Africa for medical management. The source of her infection remains undetermined. Three cases involved secondary spread of infection from the first patient, and 1 tertiary infection occurred.

## The Outbreak

**Figure 1:**
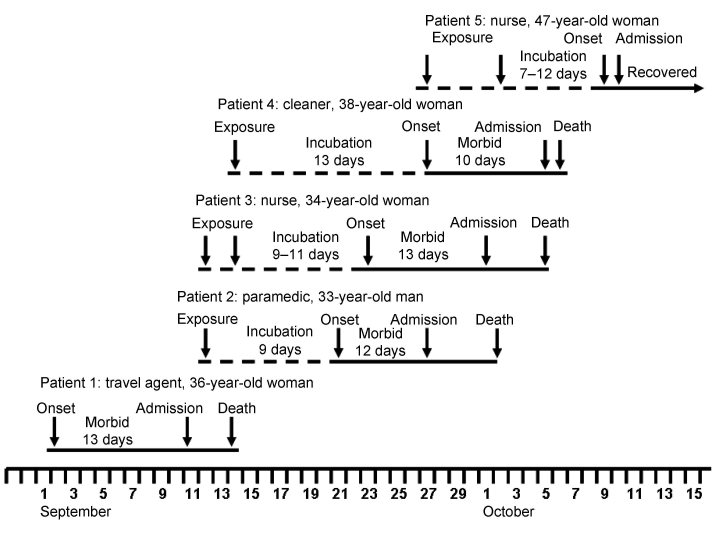
Epidemic curve showing, as appropriate, dates of exposure to infection, onset of illness, admission to hospital, and death or recovery of 5 patients involved in an outbreak of infection with a novel arenavirus, southern Africa, 2008.

Patient 1 (Figure 1) was a travel agent who lived on an agricultural smallholding on the outskirts of Lusaka, Zambia, where she kept horses, dogs, and cats. She occasionally encountered dormice in her home, and evidence of rodent activity was found in the stables. She had visited a tourist camp on the Zambezi River on July 30 and 31, 2008, and participated in a polocrosse tournament on August 1 and 2 (but neither event occurred within the known incubation period of arenavirus infections from the date of onset of her illness). On August 30, she was cut on the shin by broken glass from a dropped bottle. Severe headache and malaise developed in the patient on September 2. On September 4, she traveled by air to attend a wedding in South Africa and was taking medication for suspected influenza. Diarrhea and vomiting developed in 4 of 110 guests who attended the wedding on September 6. Patient 1 was not affected, but she reported feeling cold and took breaks from dancing to warm herself at a fireplace. On returning to Lusaka on September 7, she was exhausted and experienced an attack of diarrhea and vomiting.

She remained ill in bed on September 8. The following day she had fever, severe chest pain, and sore throat and was treated for food poisoning and influenza with antiemetic, antipyretic, and analgesic medications and a cephalosporin antimicrobial drug. On September 10, she felt slightly better, but overnight a rash developed that extended from her toes to her cheeks, as did severe myalgia and facial swelling. She was treated at a clinic on September 11 for a presumed allergic reaction to the cephalosporin, was discharged, and then readmitted that evening with severe sore throat. She was moved to a hospital for stabilization on September 12 and then evacuated by air to South Africa. During the flight, the attending physician and paramedic were potentially exposed to infection through nebulization, suctioning, and manual ventilation of the patient.

When she arrived at a private hospital in Johannesburg, her pupillary and corneal reflexes were absent, and she had cerebral edema (confirmed by computed tomography scan), acute respiratory distress syndrome, and deteriorating renal function. She had thrombocytopenia, granulocytosis, and raised serum alanine and aspartate transaminase levels. The observation of an apparent eschar on her right foot prompted treatment for rickettsiosis. Despite intensive care, including hemodialysis, she died on September 14. No autopsy was performed, and the body was returned to Zambia for cremation.

Patient 2 (Figure 1) was the paramedic who attended patient 1 during the air evacuation to Johannesburg. He had onset of illness on September 21 with headache, myalgia, and fever, and on September 24 he was admitted to a hospital in Lusaka. On September 27, he was evacuated to the same hospital in Johannesburg as patient 1, and on the following day the link between the 2 was identified and a presumptive diagnosis of viral hemorrhagic fever was made. Patient 2 died October 2. Tracing of contacts of the 2 patients was instituted, and it was found that a nurse, patient 3 (Figure 1), who had attended and cleaned the body of patient 1, became ill on September 23 while on leave. She was admitted to a private hospital west of Johannesburg on October 1, where she died October 5.

**Figure 2:**
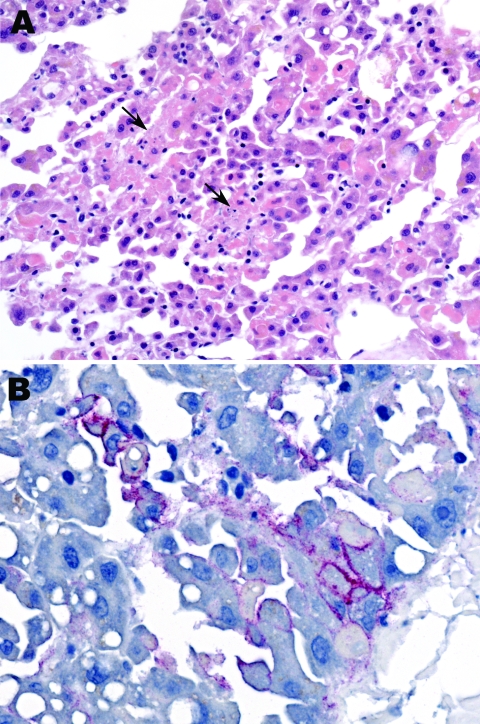
Liver biopsy specimen from patient 2 showing focal hepatocyte necrosis (arrows) without prominent inflammatory cell infiltrates (A) and Lujo virus antigens (red) distributed predominantly in a membranous pattern around infected hepatocytes (B). Hematoxylin and eosin staining in panel A and immunoalkaline phosphatase staining with naphthol fast-red stain and monoclonal antibody against GP2 Lassa virus diluted 1:1,000 in panel B. Original magnifications ×50 (A) and ×100 (B).

Blood samples were collected from patient 2 on September 29 and 30 and from patient 3 on October 3. The samples were screened at the National Institute for Communicable Diseases (NICD) for evidence of infection with known agents of the viral hemorrhagic fevers of Africa by reverse transcription–PCR (RT-PCR) for presence of viral nucleic acid, including 2 procedures with primers designed to detect all known Old World arenaviruses (*1*,*2*). Samples were also tested by ELISA for antibodies, without positive results, and injected into Vero cell cultures for isolation of virus. For administrative reasons, the taking of liver samples with biopsy needles and the taking of skin punch samples from the bodies of patients 2 and 3 was delayed until October 9, and by the next morning the Department of Anatomical Pathology at the University of the Witwatersrand reported observing hepatocyte necrosis and skin vasculitis compatible with viral hemorrhagic fever (Figure 2, panel A).

## Diagnosis and Contact Tracing

**Figure 3:**
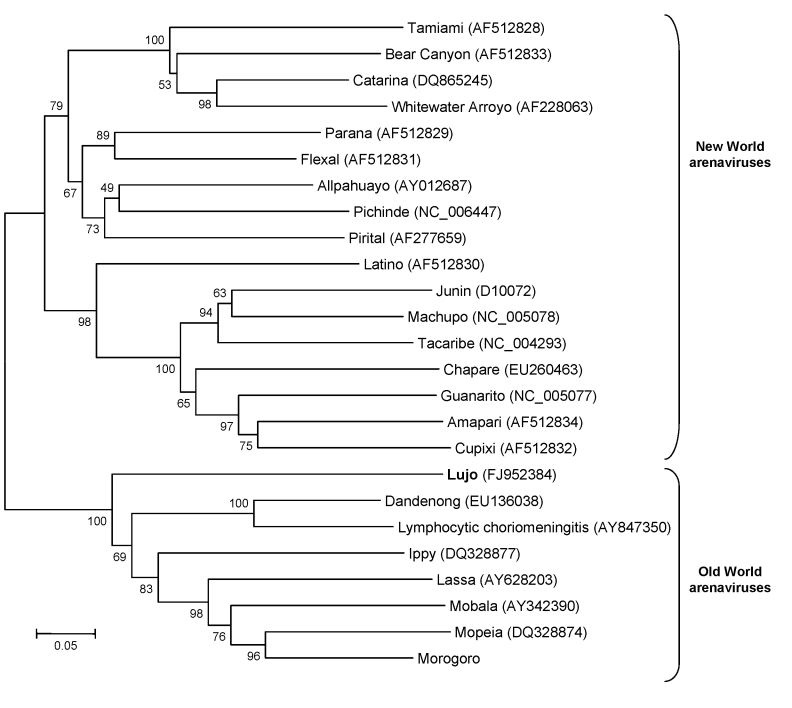
Neighbor-joining tree reconstructed by using bootstrap analysis with 1,000 pseudoreplicate datasets showing the phylogenetic relationship of known arenaviruses (data derived from GenBank) to the novel Lujo arenavirus from southern Africa (**boldface**), inferred from a 619-nt region of the 5′ end of the nucleoprotein gene. GenBank accession numbers for nucleotide sequence data are shown on the tree. Scale bar indicates 5% divergence.

Blood, liver, and skin samples sent to the Centers for Disease Control and Prevention (CDC; Atlanta, GA, USA) arrived on October 10. The following day the Infectious Disease Pathology Branch reported that immunohistochemical staining of liver and skin sections resulted in the detection of antigen with monoclonal antibody that was broadly cross-reactive for Old World arenaviruses, the first indication of an etiologic diagnosis (Figure 2, panel B). Consequently, the same arenavirus RT-PCR procedures as above were applied to liver extracts and produced positive results at NICD and the Special Pathogens Branch, CDC. One RT-PCR procedure yielded ≈300-bp glycoprotein gene product (*1*), and the other yielded ≈1,000-bp nucleoprotein gene product (*2*). Nucleotide sequencing of the PCR products and phylogenetic analysis performed as described (*3*) but using MEGA 4.0 (*4*) showed that a novel arenavirus, since named Lujo virus (*5*), was involved (Figure 3).

Replicate nucleic acid extracts from liver samples that were sent to Columbia University (New York, NY, USA) were subjected to 454 pyrosequencing to produce full-length genome sequences of the arenavirus, extending the preliminary findings based on partial sequence data to indicate that the new virus is related to, but distinct from, known Old World arenaviruses (*5*). The novel arenavirus was isolated in culture from the blood and liver samples of patients 2 and 3, as confirmed by immunofluorescence with polyvalent Old World arenavirus antiserum and sequencing of PCR products. However, the isolates from the blood samples took a minimum of 7 days to become detectable in culture, as compared with 3–5 days for liver samples and other blood samples tested during the investigation. Thus, it can be surmised that the viral loads were likely too low in the blood samples of patients 2 and 3 for detection of viral nucleic acid with the generic primers.

Through contact tracing, we found that a cleaner who worked at the hospital where patient 1 was treated, patient 4 (Figure 1), became ill on September 27, was admitted to a provincial hospital on October 5, and was transferred the same day to a tertiary academic hospital. She had a chronic underlying disease and died on the day after admission. Her only potential exposure to infection occurred September 14 when she cleaned the cubicle where patient 1 was treated. On October 9, a second nurse, patient 5 (Figure 1) became sick and was admitted to the hospital the following day. She had attended patient 2 and been involved in the insertion of a central venous line on September 27 before barrier nursing was instituted.

Treatment of patient 5 with the oral form of the antiviral drug ribavirin was started on October 11 with a loading dose of 30 mg/kg (2 g), followed by a projected schedule of 15 mg/kg every 6 h (1 g) for 4 days and 7.5 mg/kg every 8 h for 6 days. However, after the patient was intubated on October 12, treatment was continued through nasogastric tube until the intravenous formulation of the drug was obtained on October 17 (*6*). The patient reported improvement beginning October 27 and was discharged from the hospital on December 2 after RT-PCR did not detect viral RNA in blood and urine on 3 consecutive occasions.

The first 4 patients were initially managed without special infection control precautions, apart from the donning of surgical gloves for intubation or the taking of blood samples, and the use of impervious aprons in wards. Consequently, healthcare workers and maintenance staff such as cleaners were all potentially exposed to contaminated bedding, eating utensils, and bedpans which contained excreta and vomitus. On the other hand, no incidents occurred that could be construed as constituting specific exposure to infection, for example, needlesticks. After the possible involvement of a viral hemorrhagic fever was recognized on September 28 following the admission of patient 2 to the hospital in Johannesburg, barrier nursing procedures were instituted as successive patients were identified, but only patient 5 was managed with full precautions throughout her illness. The full precautions consisted of the donning of double surgical gloves, protective oversuits, impervious overshoes, disposable balaclavas, and N95 masks plus goggles or visors. Alternatively, the protective oversuits were replaced with surgical scrub-suits and impervious disposable gowns. No infections appeared to have occurred after the adoption of the full precautions.

The putative incubation periods for patients 2 to 5 ranged from 7 to 13 days, and the periods of illness for the 4 patients with fatal infections ranged from 10 to 13 days (Figure 1). All patients sought treatment for nonspecific febrile illness with headache and myalgia, which increased in severity over 7 days with the development of diarrhea and pharyngitis. A morbilliform rash became evident on the face and trunk in 3 Caucasian patients on day 6–8 of illness, but not in 2 African patients, and neck and facial swelling occurred in 3 patients. Four patients exhibited transient subjective improvement after ≈1 week of illness; this brief improvement was followed by rapid deterioration with respiratory distress, neurologic signs, and circulatory collapse, the terminal features in patients who died. Bleeding was not a prominent feature. However, 1 patient had a petechial rash, 1 had gingival bleeding, and another experienced the oozing of blood from venipuncture sites. Chest pain was a prominent symptom for 2 patients. All patients had thrombocytopenia on admission to the hospital (platelet count range 20–104 × 10^9^ cells/L). Three patients had leukocyte counts in the normal range, and 2 had leukopenia on admission, whereas leukocytosis developed in 4 patients during the illness. The maximum aspartate aminotransferase values recorded in the 4 patients who died from the disease ranged from 549 IU/L to 2,486 IU/L, compared with 240 IU/L in the survivor who was treated with ribavirin.

Blood samples from patients 4 and 5 were positive by RT-PCR for arenavirus nucleic acid at NICD and yielded virus in culture. Blood samples taken from patient 1 on September 12 and from patient 2 on September 25 were traced to a hospital laboratory in Lusaka and sent to CDC late in the course of the investigation. The sample from patient 1 was RT-PCR positive, and both samples yielded isolates of the novel virus in cell culture. In summary, isolation of virus was achieved from the blood samples of all 5 patients, plus liver samples from patients 2 and 3, at NICD, CDC, or both (Table).

**Table:** Summary of diagnostic RT-PCR and virus isolation studies on 5 novel arena virus–infected patients, southern Africa, 2008*

Patient no.	Onset of illness	Date sampled	Day of illness sampled	Sample type	RT-PCR	Virus isolation
1	Sep 2	Sep 12	11	Blood	+	+
2	Sep 21	Sep 25	5	Blood	–	+
2	Sep 21	Sep 29	9	Blood	–	+
2	Sep 21	Sep 30	10	Blood	–	+
2	Sep 21	Oct 9	12†	Liver	+	+
3	Sep 23	Oct 3	11	Blood	–	+
3	Sep 23	Oct 9	13†	Liver	+	+
4	Sep 27	Oct 6	10	Blood	+	+
5	Oct 9	Oct 10	2	Blood	+	+

*Chronology of testing samples differs from the order in which they were collected from patients. RT-PCR, reverse transcription–PCR; +, positive; –, negative. †Refers to day of illness on which the patient died.

Tracing and monitoring of all contacts of known patients for 21 days from last date of contact with a patient or fomites in Zambia and South Africa failed to identify cases additional to those reported here. Antibody surveys still need to be conducted to assess the occurrence of less severe infections and to determine the distribution and prevalence of the virus. Rodent studies are also indicated, particularly in the areas frequented by patient 1, as the original source of the infection remains unknown.

## Discussion

Arenaviruses are negative-sense single-stranded RNA viruses, most of which cause chronic infection of rodents, with excretion of virus in urine. Humans become infected from contaminated food or household items. Several New World arenaviruses are associated with hemorrhagic fevers in South America, including Junin, Machupo, Sabia, Guanarito, and Chapare viruses. Old World arenaviruses include the prototype member of the family, lymphocytic choriomeningitis virus, which has a worldwide distribution and is often associated with pet rodents. Clinical manifestations range from inapparent infection to severe meningoencephalomyelitis in humans. Dandenong virus is an arenavirus related to lymphocytic choriomeningitis virus (Figure 3) that was recently isolated in Australia from patients who had received organ transplants from a deceased donor who had traveled in eastern Europe (*7*). Lassa fever virus is an Old World arenavirus associated with the multimammate mouse (*Mastomys natalensis*) that causes hemorrhagic fever, which affects large numbers of persons in West Africa. Imported cases of the disease have occurred in Europe, North America, Asia, and South Africa ( *8**;* NICD, unpub. data). The distribution of the multimammate mouse and related species extends across much of sub-Saharan Africa. Old World arenaviruses not known to be pathogenic for humans include Ippy and Mobala viruses from the Central African Republic and Morogoro virus from Tanzania (*9*). Arenaviruses have also been found in rodents in Mozambique (Mopeia virus) and Zimbabwe (*10*,*11*) as well as South Africa (NICD, unpub. data), but phylogenetic analysis using partial nucleotide sequence data indicate that these are distinct from the new virus described here, and none of these viruses have been associated with human disease in these 3 countries, despite sustained monitoring over 3 decades.

As described elsewhere (*5*), this is the first highly pathogenic arenavirus to be identified in Africa in 4 decades. This outbreak serves as a warning that pathogenic arenaviruses could be more widely prevalent in Africa than presently realized and reinforces the need for strict screening of internationally transferred patients to ensure early recognition of infectious diseases and the maintenance of appropriate infection control precautions at all times.
